# iACT4IBD: a randomised controlled trial of a brief online intervention based on acceptance and commitment therapy to improve wellbeing for adults with inflammatory bowel disease

**DOI:** 10.3389/fdgth.2025.1587765

**Published:** 2025-06-26

**Authors:** Karen Lin, Alana Cavadino, Maggie Ow, Meihana Douglas, Anna Serlachius

**Affiliations:** ^1^ Department of Psychological Medicine, University of Auckland, Auckland, New Zealand; ^2^ Department of Epidemiology and Biostatistics, University of Auckland, Auckland, New Zealand; ^3^ Department of Gastroenterology and Hepatology, Auckland City Hospital and Greenlane Clinical Centre, Auckland, New Zealand; ^4^ School of Clinical Sciences, Auckland University of Technology, Auckland, New Zealand

**Keywords:** inflammatory bowel disease, acceptance and commitment therapy, online intervention, ulcerative colitis, Crohn's disease

## Abstract

**Introduction:**

Inflammatory bowel disease (IBD) is associated with high rates of depression and anxiety. Acceptance and Commitment Therapy (ACT) has demonstrated utility across different chronic health conditions, and online ACT interventions may potentially improve access to mental health support.

**Methods:**

This study was a parallel two-arm randomised controlled trial that investigated the effects a brief seven-day online ACT intervention (iACT4IBD) on psychological and physical health outcomes. We examined whether iACT4IBD could reduce depressive symptoms in adults with IBD compared to a waitlist control group at 4-weeks follow-up. Effects of iACT4IBD on levels of anxiety, stress, wellbeing, quality of life, and IBD activity were also explored. Seventy adults with IBD (Crohn's disease and ulcerative colitis) were randomly allocated to either the iACT4IBD intervention or a waitlist control group. Psychological and physical health outcomes were collected at baseline, 4-weeks and 8-weeks after baseline. Outcome measures were assessed at baseline and 4-weeks follow-up using mixed ANOVAs.

**Results:**

No between-group differences in psychological outcomes were found from baseline to 4-weeks follow-up. Crohn's disease activity scores in the intervention group were significantly lower after 4-weeks compared to the waitlist control group. Qualitative feedback suggested that the intervention was acceptable and beneficial.

**Discussion:**

There were no improvements to mental wellbeing; however, findings provided some support for the intervention improving physical health in people with Crohn's disease. Further research is needed.

**Clinical Trial Registration:**

Australian New Zealand Clinical Trials Registry (U1111-1282-2334).

## 1 Introduction

Inflammatory bowel disease (IBD) is an increasingly prevalent chronic health condition that is incurable. IBD affects approximately 6.8 million people globally (1) and approximately 20,000 people in Aotearoa New Zealand (2). IBD refers to a set of chronic, immune-mediated inflammatory disorders of the digestive system that feature recurrent remission and relapses, with Crohn's disease (CD) and ulcerative colitis (UC) as the most common forms (3). The unpredictability of IBD significantly impacts physical and emotional wellbeing and presents significant challenges in managing daily life (4).

Coping with IBD involves managing the physical symptoms of IBD (abdominal pain, blood loss through stools, fatigue, significant weight loss, and hospitalisation) and looking after one's mental and emotional wellbeing (5). Factors that influence IBD-related distress include loss of bowel control, body image issues, sexual inadequacy worries, feelings of uncleanliness, social isolation, fear of dependency, worries about not reaching one's full potential, and fear of stigmatisation (6). Studies report that anxiety and depression are common in people with IBD and are known to negatively affect quality of life and disease severity (7, 8). Collectively, these factors can considerably burden people with IBD (3).

A growing evidence base shows that a bi-directional gut-brain relationship affects the IBD pathophysiology; susceptibility to depression and anxiety increases with IBD diagnosis (9). Factors linking psychological stress to altered gut physiology via the gut-brain axis include gut dysbiosis (10) and increased intestinal permeability (11). Psychological stressors can enable alterations in the intestinal microbiota, which promote intestinal inflammation via increases in intestinal permeability (10, 11). Some fluctuation in the digestive system is tolerable; however, severe disruptions to gut microbiota can result in poorer physiological and mental health (12). Similarly, disease activity may negatively impact mental wellbeing leading to adverse effects on quality of life (13). Addressing psychological factors may be essential for managing IBD activity and maintaining wellbeing.

Evidence suggests that Acceptance and Commitment Therapy (ACT) is beneficial for reducing psychological dysfunction in long-term conditions (14), including treatment of mental and physical health in gastrointestinal diseases (15). ACT aims to cultivate the ability to focus on the present moment while committing to value-driven behaviours despite negative experiences—psychological flexibility (16, 17). Early evidence supports ACT as a flexible and effective therapy for treating IBD-related psychological distress (18–20); however, there is a dearth of studies delivering ACT digitally to IBD populations, despite the potential for psychological eHealth interventions to improve quality of life, disease management, and access to health services (21).

Digital health interventions are increasingly being used to increase psychological and self-management support for people living with chronic health conditions such as IBD. Digital health interventions can include web-based interventions, apps, chatbots and telehealth. These digital platforms provide various advantages over traditional face-to-face interventions, in particular their potential for scalability and cost-effectiveness (22). Existing IBD digital interventions focus on improving monitoring, education and self-management of IBD (23); however, the literature on digital mental health interventions for IBD is much more sparse, with most interventions being a hybrid approach combining both in-person and online psychological support (24). Few digital mental health interventions in IBD are purely self-guided, whereby there is no additional clinical support. Advantages of self-guided interventions include less reliance on clinical resources and reduced confounding (i.e., whether the potential improvements are due to the intervention or the clinical support).

This study aimed to build on previous findings from a brief ACT-based online study conducted in Aotearoa New Zealand during COVID-19, which found significant improvements in depressive symptoms in a cohort of adults with a range of chronic health conditions, including IBD (25). The current study examined the effects of a brief online ACT intervention (iACT4IBD) on levels of depression, anxiety, stress, wellbeing, quality of life, and disease activity compared to a waitlist control group. The primary hypothesis was that iACT4IBD would reduce depression scores at the 4-week follow-up compared to the waitlist control group. The secondary hypotheses were that iACT4IBD would improve levels of anxiety, stress, wellbeing, quality of life, and disease activity compared to a waitlist control group at 4-weeks follow-up.

### 1.1 Significance of this study

Mental health and wellbeing services are increasingly moving toward self-care models and are commonly aided by digital technologies (26). Digital health services help overcome financial and access barriers associated with traditional psychotherapy (27), and people with IBD indicate that digital health services are acceptable and convenient methods of care (28). Evidence for online ACT-based treatments is still emerging, but existing studies report promising results from online ACT interventions treating different chronic health conditions (25, 29–33), suggesting that ACT content may be appropriately distilled and learned online. There are currently no published studies delivering ACT through online mediums with a focus on the IBD population. As the first study to examine a *brief* online ACT-based intervention specifically tailored for adults living with IBD, the use of this intervention cannot yet be recommended based on these results; however, this study provides important insights into the role of digital health services for supporting psychological health in people with IBD.

## 2 Materials and methods

### 2.1 Design

This was a parallel two-arm randomised controlled trial (RCT) that examined the effects of a brief seven-day online ACT intervention (iACT4IBD) on psychological and physical health outcomes in adults with IBD living in Aotearoa New Zealand compared to a waitlist control group. This study was granted ethical approval by the Health and Disability Ethics Committees (2022 EXP 13276), locality approval from Te Whatu Ora, Auckland District Health Board, and prospectively registered on the Australian New Zealand Clinical Trials Registry (U1111-1282-2334). This study was conducted following the principles within the Helsinki Declaration (34) and The Consolidated Standards of Reporting Trials (CONSORT) (35) guidelines.

### 2.2 Sample size

Using the effect size from a similar previous study (25), we estimated the required sample size for a large effect size of ƞ_p_^2^ = 0.17 to explore the interaction between time and group in depression from baseline to 4-weeks follow-up. Using a mixed analysis of variance test (ANOVA) with an alpha of .05 and power of .90, the required sample size was 54, which we increased to 70 (35 per treatment arm) to account for attrition.

### 2.3 Participants

Recruitment started in February 2023 and was completed in April 2023. Seventy participants were recruited. Eligible participants were 18 years of age or older; diagnosed with IBD (either CD or UC); living in Aotearoa New Zealand at the time of recruitment; able to understand, read, and write English; able to access an internet-connected electronic device (e.g., computer); and able to provide informed consent. Exclusion criteria included hospitalisation, treatment for a mental health condition (apart from taking antidepressants), regular mindfulness or meditation practise, or a positive result for COVID-19 at the time of recruitment. Antidepressants are thought to have a positive impact on the course of IBD (36) and are commonly used as part of long-term pain management in chronic health conditions (37). Therefore, participants taking antidepressants were not excluded from the study. Clinical characteristics and demographic variables of the participants are included in the Supplementary Tables 1, 2.

### 2.4 Procedure

A recruitment email was sent to patients enrolled in the gastroenterology services at Auckland City Hospital and/or Greenlane Clinical Centre (Te Whatu Ora-ADHB). The study was also advertised through community organisations and online communities. All data were collected and stored using REDCap—a secure web application for managing online surveys and databases (38, 39). A researcher not involved in the study completed the randomisation using a web-based protocol (https://www.randomizer.org) to generate a random number table for group allocations. The randomisation table was pre-loaded into REDCap prior to recruitment to assign participants into either the intervention or waitlist control group after they consented to be in the study. Once screening and consenting were completed, participants were given baseline questionnaires to complete, which included demographic and clinical information, and emailed their treatment condition allocation. Four weeks post-baseline, both groups received a link to complete follow-up questionnaires. After completing these questionnaires, the waitlist group received access to iACT4IBD and then were sent a final set of questionnaires to complete 8 weeks post-baseline. Participants who completed the study were entered into a draw to win an iPad mini.

### 2.5 Intervention

**Figure 1:**
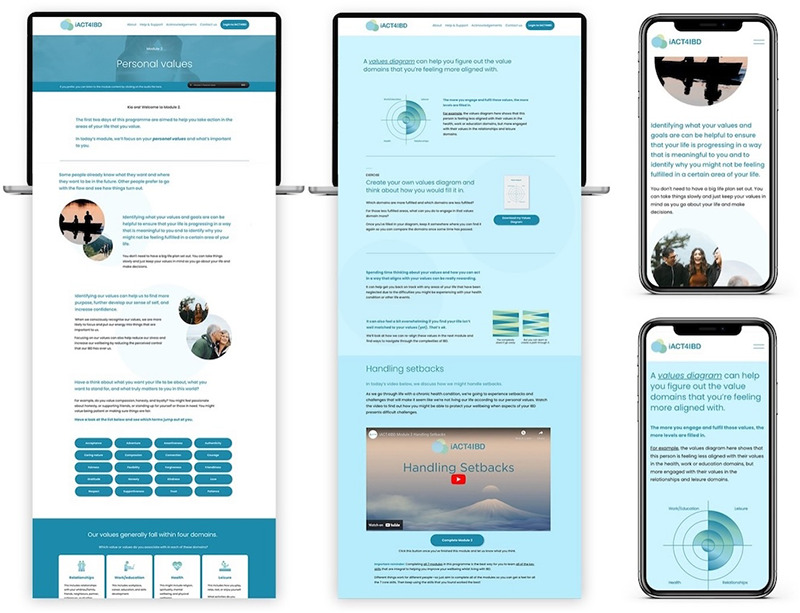
iACT4IBD module examples. Screenshots from: the ‘Personal Values’ module, iACT4IBD (https://iact4ibd.squarespace.com/).

iACT4IBD was designed using the core processes of ACT and tailored for people with IBD to complete in seven days. Daily modules consisting of approximately 20 min of ACT content. Participants could either read or listen to the intervention content online. Three reminder emails that were tailored to the IBD experience were sent to participants during the intervention to encourage them not to give up. Table 1 provides a description of the content and exercises to practice within iACT4IBD. Figure 1 shows a visual example of an iACT4IBD module.

**Table 1:** ACT4IBD module content.

Module titles	Module description
1. Introduction	Introduction to the intervention, how it works, and why it is relevant to IBD populations. Video: Recognising feelings.
2. Personal values	Recognising personal values by focusing on hopes, values, and goals that are personally important. This module explains why identifying values and goals can help to ensure one's life is progressing in a meaningful way. Exercise: Identifying personal values and using the ACT Values Bullseye to assess the balance of one's values within work/education, leisure, health, and relationship domains. Video: Handling setbacks. Noticing and naming thoughts and feelings to detach from unhelpful experiences and enable the practise of self-compassion.
3. Taking action based on what is important	Taking value-driven committed action to help people to live according to their personal values. This module explains how taking committed action helps build wellbeing by improving motivation, providing direction and clarity, and increasing satisfaction. Exercise: Setting values-oriented goals encompassing immediate (today), short (next few days), medium (next few weeks) and long-term (next few months or years) goals. Video: Looking after your body. Staying healthy with IBD by eating well, moving regularly, and getting rest.
4. Separating thoughts from feelings	Using metaphors and interactive exercises to explain cognitive defusion. This module focuses on identifying cognitive fusion and explaining the purpose and benefit of cognitive defusion. Exercises: Practising cognitive defusion by (a) creating distance from an unhelpful thought using “I'm having the thought that…”; (b) *externalising* the thought (e.g., giving it a silly voice) to un-attach from it; and (c) visually distorting the image of a thought to reduce its power. Video: Staying connected. Connecting with others for a sense of support to reduce feelings of isolation.
5. Mindfulness and the present moment	Being mindful and aware of the present moment using self-compassion and mindfulness exercises. This module explains the benefits of increasing mindfulness and focuses on ways to increase awareness of the present moment. Exercises: Building present moment awareness by (a) connecting with the senses and focusing on *noticing*; and (b) paying conscious attention to a familiar activity. Video: Mindful breathing. A brief mindful breathing exercise to support connecting with the present moment.
6. Our observing mind	Using the observing mind (and mindfulness) to defuse unpleasant thoughts and experiences. This module helps participants learn to differentiate between the *observing mind* and *thinking mind*. Exercises: Using the observing mind by (a) writing down all thoughts observed for two minutes; and (b) using mindful breathing to notice and create distance from thoughts. Video: Relaxing the mind. Practising visualisation as a relaxation technique.
7. Accepting unwanted experiences	Accepting all experiences to allow unpleasant ones to redirect focus and energy to the meaningful aspects of life. This module uses metaphoric reference to explain the impact of struggling against difficult thoughts, feelings, or symptoms, and focuses on the benefit of acceptance of challenging situations. Exercise: *Noticing* what happens during an unpleasant experience over the next week to recognise one's *reaction.* Video: Self-compassion. Engaging in self-compassion during a difficult experience by being mindful of one's thoughts, feelings and surroundings; recognising that we're not alone; and speaking kindly to oneself.

### 2.6 Measures

#### 2.6.1 Demographic and clinical data

Demographic data included age, ethnicity, gender, whether participants were currently studying, level of education, living arrangements, and relationship status. Clinical information included IBD diagnosis, age at diagnosis, years since diagnosis, comorbid medical conditions, current medications, dietary regimens to help with IBD, and other things (e.g., acupuncture or other alternative medicine treatments) to help with IBD, which they then were asked to describe in a free-form textbox.

#### 2.6.2 Depression, anxiety, and stress

Depression, anxiety, and stress were assessed with the Depression, Anxiety and Stress Scale (40). The 21-item version of this measure (DASS-21) consists of three 7-item subscales to define normal, moderate, severe, or extremely severe levels of depression, anxiety, or stress. Items are scored on a 4-point Likert scale from 0 to 3 and raw scores from each subscale are summed and then multiplied by two for a total score between 0 and 42. Higher scores define more severe psychopathology. The DASS-21 has good internal consistency (*α* = .94) (41) and has been used in various ACT-based interventions for chronic health conditions (14, 20, 42). The DASS-21 demonstrated good internal consistency in the present study (*α* = .91) at baseline.

#### 2.6.3 Wellbeing and quality of life

Wellbeing was assessed with the World Health Organisation Well-being Index (WHO-5) (43). The WHO-5 is a 5-item wellbeing measure scored on a 6-point Likert scale from 0 to 5. Raw scores are summed from the items and then multiplied by four for a total score as a percentage. Higher scores represent better quality of life. The WHO-5 is a reliable and valid measure of subjective wellbeing that has been used to evaluate different long-term conditions (44). The WHO-5 demonstrated good internal reliability in the present study (*α* = .85) at baseline.

Health-related quality of life was measured using the Short Inflammatory Bowel Disease Questionnaire (SIBDQ) (45). The SIBDQ is a 10-item scale measuring quality of life in four domains important for IBD: physical, social, emotional, and systemic. Each item is scored using a 7-point Likert scale ranging from 1 to 7 and summed for a total health-related quality of life score ranging from 10 to 70. Score ranges define slightly (10–45), moderately (45–60), or severely (60–70) worsened quality of life (46). The SIBDQ demonstrated good internal reliability (*α* = .78) and a test-retest reliability (.65) in patients with stable CD (45) and has been validated for the UC population (47). The internal reliability of the SIBDQ in the current study was .76 at baseline.

#### 2.6.4 Disease activity

CD activity was assessed using the Harvey-Bradshaw Index (HBI) (48). The HBI was developed to assess response to IBD treatments and evaluate long-term progress (48). The index uses five variables to score the occurrence and severity of disease activity: general wellbeing, abdominal pain, number of liquid stools per day, abdominal mass, and complications. CD activity scores on the HBI can range from 0 to over 16 to define clinical remission (<5), mild disease (5–7), moderate disease (8–16), and severe disease (>16) (48, 49).

UC disease activity was measured using the Simple Clinical Colitis Activity Index (SCCAI) (50). The SCCAI is a validated 5-item symptom-based index of clinical criteria for UC (50). The index assesses symptoms experienced over the past three days: bowel frequency, urgency of defecation, blood in the stool, general wellbeing, and extracolonic features. Items are scored based on severity or frequency, with higher points indicating more severe symptoms. A score of 5 or more on the SCCAI indicates disease activity or relapse (51) UC scores on the SCCAI have been used to define clinical remission (≤2), mild disease (3–5), moderate disease (6–11), and severe disease (≥12) (52).

#### 2.6.5 Acceptability and adherence

Participants self-reported their completion of and feedback for each module, with a free-form textbox collecting data on the acceptability of iACT4IBD. An acceptable adherence rate was defined *a priori* as the completion of at least five modules.

### 2.7 Data analyses

This study used an intention-to-treat approach for the quantitative analyses of all outcome measures. A series of mixed ANOVAs (2 groups by 2 time points) were used to compare change over time from baseline to 4-weeks after baseline between the intervention group (*n* = 22) and the waitlist control group (*n* = 28). Quantitative analyses were not conducted at the 8-week follow-up due to insufficient data collected; however characteristics and outcome measures of the sample at 8-weeks follow-up are described; refer to Supplementary Table 3.

Adherence was measured by the number of modules participants reported as complete. Free-form qualitative feedback was analysed and coded using directed content analysis (53) and mapped onto an adapted version of a usability framework for digital health technologies (54). The adapted usability framework consisted of three categories to code users' feedback: (1) usability, (2) design, and (3) clinical role.

## 3 Results

**Figure 2:**
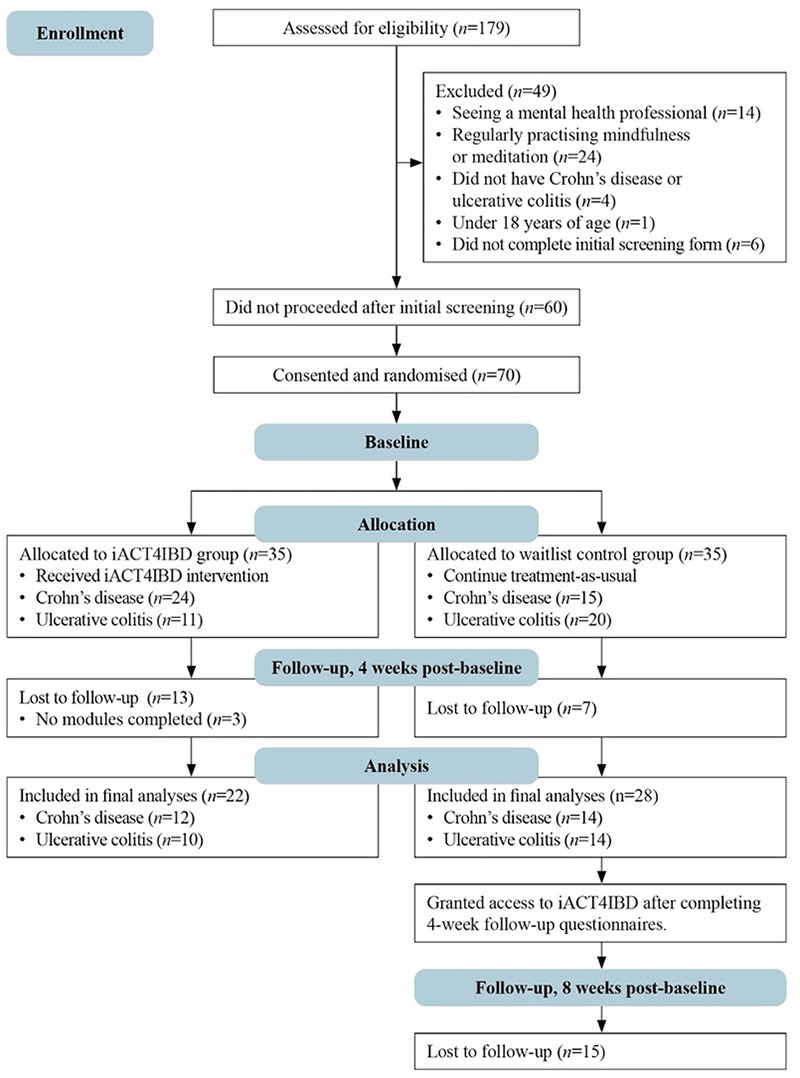
CONSORT diagram of participant flow.

Figure 2 shows the CONSORT diagram. The demographic and clinical characteristics of study participants are displayed in the Supplementary Tables 1, 2.

### 3.1 Demographics and clinical characteristics

Out of 179 people that were assessed for this study, 70 adults living with IBD (CD or UC) were eligible, consented, and randomised to either the intervention or waitlist control group. Participant ages were between 18 and 82 years, with a mean age of 39.9 years (*SD* = 14.0). More than half of the sample were female (74%). Most of the sample identified as New Zealand European (86%), were not currently studying (87%), and were either employed full-time (56%) or part-time (21%). Education levels were mixed, and many participants were either living with a partner (40%) or with family (39%). Most participants were in some form of a relationship, with 47% being married.

The age at diagnosis ranged from 3 to 70 years, with a mean diagnosis age of 28.1 years (*SD* = 11.8). The mean number of years since diagnosis was 11.7 (*SD* = 9.4). More than half of the participants (60%) did not have another medical condition. Some people had specific dietary regimens to help manage their IBD (27%) but most (86%) were not doing anything else to help manage their IBD. Most people were not taking antidepressants (79%), however 21% of participants were taking antidepressants while in this study.

### 3.2 Baseline

At baseline, normal range scores were reported by participants for depression (63%), anxiety (60%), and stress (36%). Wellbeing at baseline was 48.11 (*SD* = 18.59). The mean quality of life score was 48.31 (*SD* = 9.03). Baseline scores indicated that 18% of the participants with CD and 23% of the participants with UC were in remission, while the remaining participants had varying degrees of active disease. The intervention group consisted of 24 participants with CD and 11 participants with UC and the waitlist group consisted of 15 participants with CD and 20 participants with UC. Table 2 shows the outcome measures at baseline.

**Table 2:** Mental and physical health outcomes at baseline.

Outcome variable	Waitlist (control, *n* = 35) *n* (%)	Intervention (iACT4IBD, *n* = 35) *n* (%)	Total sample (*N* = 70) *N* (%)
Depression score^1^^,^^a^ *M(SD)*	8.00 (8.91)	9.71 (6.72)	8.86 (7.88)
Normal	24 (69%)	20 (57%)	44 (63%)
Mild	6 (17%)	6 (17%)	12 (17%)
Moderate	1 (3%)	5 (14%)	6 (9%)
Severe	1 (3%)	4 (11%)	5 (7%)
Extremely severe	3 (9%)	0 (0%)	3 (4%)
Anxiety score^2^^,^^a^ *M(SD)*	6.86 (6.31)	7.03 (6.31)	6.94 (6.27)
Normal	21 (60%)	21 (60%)	42 (60%)
Mild	5 (14%)	5 (14%)	10 (14%)
Moderate	4 (11%)	3 (9%)	7 (10%)
Severe	3 (9%)	3 (9%)	6 (9%)
Extremely severe	2 (6%)	3 (9%)	5 (7%)
Stress score^3^ *M(SD)*	12.51 (8.41)	14.34 (7.07)	13.43 (7.77)
Normal	16 (46%)	9 (26%)	25 (36%)
Mild	11 (31%)	18 (51%)	29 (41%)
Moderate	6 (17%)	6 (17%)	12 (17%)
Severe	2 (6%)	2 (6%)	4 (6%)
Extremely severe	0 (0%)	0 (0%)	0 (0%)
Wellbeing score^4^ *M(SD)*	51.66 (19.34)	44.57 (17.36)	48.11 (18.59)
Quality of life score^5^ *M(SD)*	48.20 (9.94)	48.43 (8.17)	48.31 (9.03)
Slightly worsened	16 (46%)	12 (34%)	28 (40%)
Moderately worsened	15 (43%)	22 (63%)	37 (53%)
Severely worsened	4 (11%)	1 (3%)	5 (7%)
Crohn's activity score^6^^,^^a^^,^^b^ *M(SD)*	6.67 (3.33)	7.18 (3.93)	6.97 (3.66)
Remission	3 (20%)	4 (17%)	7 (18%)
Mild disease	7 (47%)	13 (54%)	20 (51%)
Moderate disease	5 (33%)	6 (25%)	11 (28%)
Severe disease	0 (0%)	1 (4%)	1 (3%)
Ulcerative colitis score^7^^,^^a^^,^^b^ *M(SD)*	5.15 (2.48)	4.18 (2.18)	4.81 (2.39)
Remission	4 (20%)	3 (27%)	7 (23%)
Mild disease	6 (30%)	5 (46%)	11 (36%)
Moderate disease	10 (50%)	3 (27%)	13 (42%)
Severe disease	0 (0%)	0 (0%)	0 (0%)

^a^ Percentages do not add up to 100 due to rounding.

^b^ Numbers add up to 35 when combining CD and UC for each group since participants had either CD or UC.

### 3.3 4-weeks follow-up

Table 3 shows results of mixed ANOVAs, conducted to investigate whether participants in the intervention group would show reductions in depressive symptoms, anxiety and stress, and improvements in wellbeing, quality of life and disease activity from baseline to 4-weeks follow-up compared to the waitlist control group. Results showed that the intervention did not significantly improve depressive symptoms or any of the secondary outcome measures at 4-weeks follow-up. However, the group difference at four weeks was significant for CD activity *F*(1, 22) = 7.05, *p* = .014, ƞ_p_^2^ = 0.11, indicating that there was a significant difference in CD activity scores between participants allocated to the intervention compared to the waitlist control group at 4-weeks follow-up.

**Table 3:** Comparisons of mental and physical health outcomes at 4-weeks follow-up.

Outcome variable	iACT4IBD intervention group mean (*n* = 22) *M* (*SD*)	Waitlist control group mean (*n* = 28) *M* (*SD*)	Marginal mean difference iACT4IBD versus waitlist (95% CI)	*p*-value for mean difference	*p*-value for group by time interaction	Partial eta squared (ƞ_p_^2^)
Depression score						
Baseline	8.00 (6.02)	8.36 (9.80)	−0.36 (−5.14–4.43)	.881	.763	.00
Four-weeks	8.55 (9.30)	9.50 (11.22)	−0.96 (−6.93–5.02)	.749		
Anxiety score						
Baseline	5.64 (5.40)	6.57 (6.32)	−0.94 (−4.34–2.47)	.583	.093	.06
Four-weeks	4.91 (4.52)	7.71 (6.90)	−2.81 (−6.23–0.62)	.11		
Stress score						
Baseline	13.27 (7.34)	12.07 (8.67)	1.20 (−3.45–5.85)	.606	.091	.06
Four-weeks	11.73 (6.42)	13.79 (10.10)	−2.06 (−7.03–2.92)	.410		
Wellbeing^a^						
Baseline	11.55 (4.59)	13.18 (4.85)	−1.63 (−4.35–1.08)	.232	.619	.01
Four-weeks	11.41 (5.44)	12.50 (5.57)	−1.091 (−4.25–2.07)	.491		
Quality of life score						
Baseline	51.77 (6.74)	49.11 (9.54)	2.67 (−2.16–7.49)	.273	.646	.00
Four-weeks	51.05 (6.67)	47.68 (10.37)	3.37 (−1.76–8.49)	.193		
CD activity score						
Baseline^b^	5.83 (2.55)	6.83 (3.19)	−1.00 (−3.44–1.44)	.405	.117	.11
Four-weeks^c^	4.75 (2.96)	7.83 (2.73)	−3.08 (−5.49 to −0.68)	.014*		
UC score						
Baseline^d^	4.30 (2.26)	5.00 (2.45)	−0.70 (−2.74–1.34)	.484	.443	.03
Four-weeks^e^	3.90 (2.23)	3.86 (2.32)	0.04 (−1.92–2.00)	.964		
Currently experiencing a flare *n* (%)						
Baseline	5 (23%)	5 (18%)		.964		
Four-weeks	3 (14%)	6 (21%)		.495		

^a^ Raw scores.

^b^ Intervention group (*n* = 24), Waitlist group (*n* = 15).

^c^ Intervention group (*n* = 12), Waitlist group (*n* = 12).

^d^ Intervention group (*n* = 11), Waitlist group (*n* = 20).

^e^ Intervention group (*n* = 10), Waitlist group (*n* = 14).

* *p* < .05.

### 3.4 8-weeks follow-up

Supplementary Table 3 shows the characteristics and outcome measures of the sample at 8-weeks follow-up. Thirteen people (3 with CD and 10 with UC) aged 22–82 years (mean age = 43 years) in the waitlist group completed the 8-week follow-up. The mean time since diagnosis for participants who completed the 8-week follow-up was 11.6 years. Fifteen people (9 with CD and 6 with UC) aged 25–74 years (mean age = 44 years) in the waitlist group did not complete the 8-week follow-up. The mean time since diagnosis for participants who did not complete the 8-week follow-up was 13.3 years.

### 3.5 Acceptability and adherence

**Figure 3:**
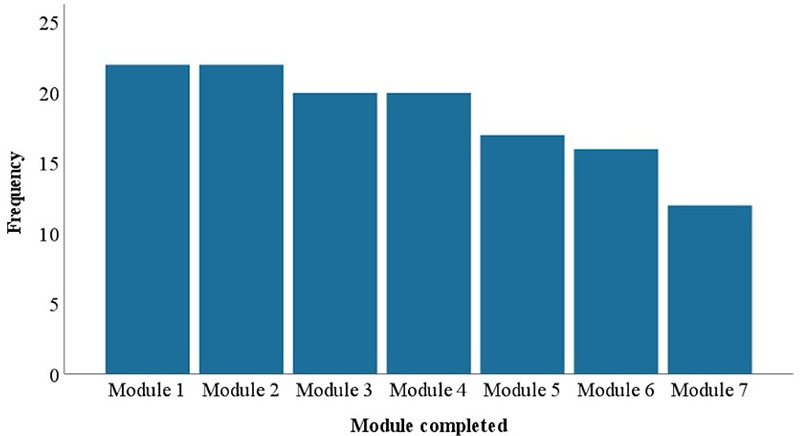
Number of iACT4IBD modules completed at 4-weeks follow-up.

Adherence to the intervention was examined via self-report in the intervention group. All participants reported completing at least three modules, while 12 participants reported completing all modules and 16 participants met the *a priori* threshold for adherence, completing at least five modules, regardless of the order of completion (see Figure 3).

Feedback was mostly positive for the iACT4IBD intervention, with findings indicating that the content delivered in the intervention was considered beneficial by participants. The open-format feedback provided valuable information on key aspects of the intervention relating to its usability, design, and clinical relevance for improvement and refinement to meet the needs of people living with IBD.

Clinically-relevant aspects that participants enjoyed included learning ACT strategies that helped them recognise and separate from negative thoughts and learning about self-compassion. Feedback showed that the intervention content was concisely presented, easy to engage with, and easy to understand, and some participants mentioned liking the mindfulness and visualisation exercises. Suggestions for improvement included providing more explanation into the activities for context, distinguishing between positive and negative feelings when discussing how to observe and recognise them, elaborating on the science underpinning the benefits of stress reduction for IBD symptoms, and acknowledging the diverse ways that people might cope and feel when living with IBD.

Regarding design aspects of the intervention, most participants commented positively about the audio and video features. The majority of participants found the videos interesting, helpful, well-paced, and enjoyable and noted the calm audio commentary as a positive aspect of the intervention. Participants also reported that the website content was presented in an easy-to-understand manner, and they thought that the prompts provided for practising self-compassion and defusion were helpful.

Usability aspects of the intervention were mainly related to a desire for further explanation into the activities within the modules. Participants noted that the values activity required clarification and more explanation for engaging with the values diagram. Participants also mentioned that they expected some aspects of the website to be interactive when they were not (e.g., words that resembled buttons but were not interactive).

Table 4 provides an overview of the open-format feedback responses.

**Table 4:** Overview of the open-format feedback responses.

Category	Sub-category	Frequency	Example quotes
Usability aspects	Descriptions	6 out of 10 responses	“From a practical point of view I think you need to explain a bit more clearly how to create the values diagram. I managed in the end…I liked this module very much (in spite of the difficulty) as it felt like a real fundamental review of why I am here and what I might achieve. It has left me feeling positive and optimistic - a feeling that it is within my power to change my life for the better.” (Male, 53 years)
Usability aspects	Controls	2 out of 10 responses	“One technical issue is that when you click on the different values that are listed, it just reloads the page.” (Female, 30 years)
Design features	Video features	16 out of 31 responses	“I found the video helpful and exactly how I feel at times.” (Female, 49 years)
Design features	Language used	12 out of 31 responses	“Well put together. Content is easily to engage with and understand.” (Male, 47 years)
Design features	Sound or audio	5 out of 31 responses	“Nice calm voice to listen to the presentation.” (Female, 66 years)
Clinically related feedback	Psychoeducation	26 out of 53 responses	“Great video. Easy to understand and relate to. Particularly liked the grounding and body work e.g., Holding hands, to separate from the mind, as so often we just focus on our negative thoughts and ruminate, and don't see ourselves as separate to our thoughts.” (Female, 37 years)
Clinically related feedback	Skill learning	29 out of 53 responses	“It was very helpful to learn some sentences of how we can talk to ourselves to enable self-compassion… I liked the tips on being present in the moment and being kind to ourselves.” (Female, 41 years)
Clinically related feedback	Clarification of content	9 out of 53 responses	“I think some further steps/instructions would have been useful for the values activity. I wasn't sure how many values to pick, if they can be different between the different areas (e.g., between work and leisure) and how the shading works?” (Female, 25 years)

## 4 Discussion

Research on digitally delivered ACT interventions for IBD populations is still developing. Although no significant improvements in mental wellbeing were found, findings from the current study introduce some novel and interesting avenues to pursue in future IBD research related to physical and psychological aspects of living with the chronic health condition. The current randomised controlled trial of a brief seven-day online ACT-based intervention suggests that iACT4IBD was acceptable to adults with IBD. Qualitative feedback indicated that the online intervention was concise, comprehensible, and easy to engage with.

High-quality RCTs testing ACT-based interventions for chronic health conditions are limited but growing (14); however, IBD studies using web-delivered ACT treatments are scarce. The current results are consistent with previous findings from ACT-based studies that reported non-significant differences in depressive symptoms in IBD populations (19, 55), but in contrast to other findings that found improvements in depressive symptoms (20), anxiety (19, 55) and stress (18, 20, 55) from ACT interventions. Our study builds on the findings from a previous pilot study conducted in Aotearoa New Zealand during COVID-19 of a brief online ACT-based intervention (25), which found significant improvements in depressive symptoms in adults with different chronic health conditions, including IBD. This previous study was conducted during a period of heightened stress and health anxiety, which may have contributed to the significant between-group differences in depressive symptoms. Possible factors that impacted the current findings are outlined below.

Low levels of psychopathology at baseline in the present study suggest a *floor effect* (56) wherein it was difficult to find significant changes due to largely normal-range depression and anxiety levels at baseline, which was previously noted as a challenge for web-delivered ACT-based studies addressing mental wellbeing (26). Earlier research also reported there may be subtle differences in cognitive appraisal between CD and UC populations (57), but how cognitive appraisals differ within IBD subtypes to subsequently impact physical and psychological health is not well understood. It is important to note that the group difference in CD activity reported at follow-up may have been due to a slight non-significant deterioration in the waitlist control group over time rather than the intervention since there was an absence of significant improvement over time in the intervention group and no interaction between time and group. There may also be a chance of a false positive given the unadjusted statistical tests within the present study. Moreover, design factors may also have a role in the present findings. While the brevity of iACT4IBD modules can be considered more scalable and cost-efficient compared to face-to-face interventions that demand clinician input, module brevity may also have impacted the opportunity to fully develop ACT skills.

Beyond factors impacting the current results are theoretical and clinical implications that warrant discussion. Firstly, the current findings suggest that iACT4IBD may be better suited for cultivating psychological change when there are higher levels of psychopathology. Secondly, interactions between psychological and physical health outcomes have previously been reported to be different in people with CD vs. UC (57, 58). Previous studies have identified subtle differences in how personality interacts with disease consequences in CD and UC, suggesting that psychobiological interactions may be different between the two IBD subtypes (57). Since CD is considered a more serious IBD subtype than UC due to a greater proportion of the gastrointestinal tract potentially being affected by disease activity (57), it is conceivable that gut-brain processes might be different in people with CD compared to UC, enabling greater utility of ACT-based approaches in CD populations when IBD symptoms are more severe. Thirdly, growing evidence shows that ACT skills can be effectively taught and delivered digitally to people with various chronic health conditions (16). Delivering psychological treatments online may help to improve reach and expand access to mental health support for people with IBD.

When considering the implications for future research, an important consideration is both the informational and emotional needs of people living with IBD. Several studies have found that people with IBD crave information on a diverse range of topics relating to managing IBD, including education about treatment, medication side-effects, and other important topics related to IBD management, such as diet, stress management and psychological support (59, 60). These consistent patient concerns demonstrate the lack of a holistic approach to managing IBD in routine care and highlight the potential for tailored digital health interventions to not only address the diverse informational needs of people living with IBD, but also their emotional needs. One aspect that we aim to address in future iterations of iACT4IBD is adding more information regarding treatment and self-management challenges, including information on diet, sleep and exercise in IBD, which are likely to impact on both physical and mental health outcomes. We also aim to replicate the trial in a cohort of newly diagnosed adults with IBD, to maximise the impact on mental health outcomes.

This study has several strengths to acknowledge. One key strength was that this was the first randomised controlled trial to examine the effects of a brief online ACT-based intervention tailored for people living with IBD in the world, providing a valuable contribution to the literature on ACT interventions for people with IBD. iACT4IBD also included different content delivery formats and used responsive website design to maximise scalability and accessibility to people with IBD living in different regions. Shorter interventions may be more feasible and potentially more acceptable for people with chronic health conditions since they are easier to deliver in traditional healthcare settings and may be more cost and resource effective for the individual and provider (42). Moreover, brief digital ACT-based interventions may facilitate access to treatment and a willingness to participate and be less distressing for IBD outpatients who have difficulty attending traditional face-to-face treatment (42).

The limitations in this study present several interesting avenues for future studies to pursue. The greatest limitation in this RCT relates to participant retention, a similar challenge reported by other RCTs focusing on the IBD population (20, 61). Situational factors may have contributed to the attrition rate in this study since the recruitment period coincided with two closely-timed severe weather events in Aotearoa New Zealand, potentially leaving participants feeling less motivated to engage with iACT4IBD due to displacement, property damage, or access to the internet. Adaptive coping strategies for IBD may have been especially difficult during this period and participants may have been disproportionately fatigued—an increasing problem for people living with IBD (62). It has previously been noted that longer ACT interventions (defined as more than five sessions) may be more beneficial for people with chronic health conditions due to the long-term and sometimes severe nature of many health conditions (14). Extending the length of the ACT intervention may allow participants more time to learn the required coping skills since an insufficient dosage of ACT was previously linked to non-significant improvements in mental health (63). However, longer self-delivered interventions may present motivational challenges when difficult IBD symptoms occur. Motivational difficulties have been associated with self-led ACT interventions; therapist-led sessions were preferred for the support, enhanced motivation, and improved comprehension of content (64). Hence, blended online ACT approaches may be an interesting area to pursue in future IBD studies.

Another limiting factor related to the absence of stratified sampling to obtain an ethnically varied and gender-diverse sample with clinically significant levels of psychopathology, which resulted in a largely homogenous sample of New Zealand European women with relatively normal-range psychological function. The sampling strategy and the delivery of the intervention solely in English made generalisability of the results more challenging outside of English-speaking adults. Screening processes required participants to be fluent in English, which could have potentially excluded a proportion of the IBD population if English was not their first language. Additionally, some intervention concepts may not have resonated with certain participants due the limits of English within different cultural contexts within New Zealand. However, since most participants had already been living with IBD for many years, they may have had the time to develop enduring ways to cope with IBD, resulting in limited utility of this intervention. Future studies could examine a similar intervention offered in more languages in people who are newly diagnosed with IBD, have higher rates of psychological distress at baseline, and use a larger and more diverse sample to explore age and ethnicity effects. Additionally, the modest adherence rate in the current study also presents opportunities to include more effective adherence measures, such as automated forms of digital tracking (e.g., web analytics), to better understand when participants ceased participation in the intervention. Due to the novelty of this study, there were a number of unpredictable limitations, which suggests the dire need for more research in this area. Future researchers may also consider exploring possible mechanisms (e.g., psychological flexibility or self-care behaviours) for psychological change.

## 5 Conclusion

This study extends the current understanding of the role of digital mental health interventions for adults with IBD and has addressed a noteworthy gap in the literature by examining the effects of a brief ACT-based intervention delivered entirely online for an IBD population. While no significant effects on mental wellbeing were found, the current study presented interesting avenues for future research on online ACT-based interventions for people living with IBD, particularly Crohn's disease; however further investigation is needed to validate and expand on these results. Future studies can improve the sampling approach, ACT dosage provided, and the follow-up of digitally delivered ACT interventions for people with IBD.

## Data Availability

The datasets presented in this article are not readily available because participants of this study did not agree to share their data publicly, so supporting data are not available. Requests to access the datasets should be directed to Anna Serlachius a.serlachius@auckland.ac.nz.

## Footnotes

^1^Depression score ranges (normal = 0–9; mild = 10–12; moderate = 13–20; severe = 21–27; extremely severe = 28–42).

^2^Anxiety score ranges (normal = 0–6; mild = 7–9; moderate = 10–14; severe = 15–19; extremely severe = 20–42).

^3^Stress score ranges (normal = 0–10; mild = 11–18; moderate = 19–26; severe = 27–34; extremely severe = 35–42).

^4^Wellbeing score ranges (0 = absence of wellbeing, 100 = maximal wellbeing).

^5^Quality of life score ranges (slightly worsened = 10–45; moderately worsened = 45–60; severely worsened = 60–70).

^6^CD activity score ranges (remission < 5; mild disease = 5–7; moderate disease = 8–16; severe disease > 16).

^7^UC activity score ranges (remission ≤3; mild disease = 3–5; moderate disease = 6–11; severe disease ≥ 12).
