# Supplementary material for: iACT4IBD: a randomised controlled trial of a brief online intervention based on acceptance and commitment therapy to improve wellbeing for adults with inflammatory bowel disease
Source: Front Digit Health. 2025 Jun 26;7:1587765. doi: 10.3389/fdgth.2025.1587765 (PMC12241075; doi:10.3389/fdgth.2025.1587765)
Supplement: Supplementary file 1 [file Table1.pdf]

Clinical Characteristics at Baseline

**Clinical Characteristic**	**Intervention** (iACT4IBD, *n* = 35) *n* (%)	**Waitlist** (control, *n* = 35) *n* (%)	**Total Sample** (*N* = 70) *N* (%)
**IBD diagnosis**[b]			
Crohn's disease	24 (69%)	15 (43%)	39 (56%)
Ulcerative colitis	11 (31%)	20 (57%)	31 (44%)
**Years since diagnosis** *M(SD)*	11 (9.1)	12.3 (9.8)	11.7 (9.4)
**Age at diagnosis in years** *M(SD)*	27 (9.8)	29.1 (13.7)	28.1 (11.8)
**Currently experiencing a flare**			
No	25 (71%)	27 (77%)	52 (74%)
Yes	10 (29%)	8 (23%)	18 (26%)
**Comorbid medical conditions**			
No	21 (60%)	21 (60%)	42 (60%)
Yes	14 (40%)	14 (40%)	28 (40%)
**Number of comorbid medical conditions**			
None	21 (60%)	21 (60%)	42 (60%)
One	7 (20%)	8 (23%)	15 (21%)
Two	2 (6%)	4 (11%)	6 (9%)
Three	4 (11%)	2 (6%)	6 (9%)
Five	1 (3%)	0 (0%)	1 (1%)
**Number of current medications** *M(SD)*	3.43 (2.56)	3.09 (2.87)	3.26 (2.71)
**Current medications**[a]			
Immunosuppressants	27 (77%)	19 (54%)	46 (66%)
Anti-inflammatory	10 (29%)	15 (43%)	25 (36%)
Anti-depressants	5 (14%)	10 (29%)	15 (21%)
Pain	1 (3%)	2 (6%)	3 (4%)
Cardiovascular	2 (6%)	4 (11%)	6 (9%)
Vitamin or supplements	10 (29%)	6 (17%)	16 (24%)
Other	21 (60%)	15 (43%)	36 (51%)
**On a specific diet to help with IBD**			
No	27 (77%)	24 (69%)	51 (73%)
Yes	8 (23%)	11 (31%)	19 (27%)
**Doing other things to help with IBD**			
No	31 (89%)	29 (83%)	60 (86%)
Yes	4 (11%)	6 (17%)	10 (14%)

[a] Percentages do not add up to 100% because participants may be taking multiple medications.

[b] Includes data from two participants who responded with UC at baseline and CD at follow-up.
