# Supplementary material for: iACT4IBD: a randomised controlled trial of a brief online intervention based on acceptance and commitment therapy to improve wellbeing for adults with inflammatory bowel disease
Source: Front Digit Health. 2025 Jun 26;7:1587765. doi: 10.3389/fdgth.2025.1587765 (PMC12241075; doi:10.3389/fdgth.2025.1587765)
Supplement: Supplementary file 2 [file Table2.pdf]

Demographic Variables at Baseline

**Demographic Variable**	**Waitlist** (control, n = 35) n (%)	**Intervention** (iACT4IBD, n = 35) n (%)	**Total Sample** (N = 70) N (%)
**Age in years** *M(SD)*	41.5 (16.0)	38.3 (11.6)	39.9 (14.0)
**Gender**[b]			
Male	10 (29%)	7 (20%)	17 (24%)
Female	25 (71%)	27 (77%)	52 (74%)
Non-binary	0 (0%)	1 (3%)	1 (1%)
**Ethnicity**[a, b]			
New Zealand European	31 (89%)	29 (83%)	60 (86%)
Māori	1 (3%)	1 (3%)	2 (3%)
Indian	3 (9%)	1 (3%)	4 (6%)
Other	1 (3%)	6 (17%)	7 (10%)
**Currently studying**			
No	29 (83%)	32 (91%)	61 (87%)
Yes	6 (17%)	3 (9%)	9 (13%)
**Highest level of education**[b]			
Did not complete high school	2 (6%)	0 (0%)	2 (3%)
Completed high school	5 (14%)	8 (23%)	13 (19%)
Started undergraduate degree	6 (17%)	1 (3%)	7 (10%)
Completed undergraduate degree	16 (46%)	21 (60%)	37 (53%)
Completed postgraduate degree	6 (17%)	5 (14%)	11 (16%)
**Employment status**[b]			
Employed full-time	19 (54%)	20 (57%)	39 (56%)
Employed part-time	6 (17%)	9 (26%)	15 (21%)
Employed on contract/casual	3 (9%)	2 (6%)	5 (7%)
Unemployed/not working	1 (3%)	0 (0%)	1 (1%)
Homemaker	0 (0%)	2 (6%)	2 (3%)
Sickness/disability beneficiary	1 (3%)	1 (3%)	2 (3%)
Student	3 (9%)	1 (3%)	4 (6%)
Retired	2 (6%)	0 (0%)	2 (3%)
**Living arrangements**[b]			
Living alone	5 (14%)	2 (6%)	7 (10%)
Living with family	11 (31%)	16 (46%)	27 (39%)
Living with a partner	15 (43%)	13 (37%)	28 (40%)
Living with friends/flatmates	4 (11%)	4 (11%)	8 (11%)
**Relationship status**[b]			
Single	9 (26%)	7 (20%)	16 (23%)
In a relationship	3 (9%)	3 (9%)	6 (9%)
De facto (not married but living together)	8 (23%)	6 (17%)	14 (20%)
Married	14 (40%)	19 (54%)	33 (47%)
Widowed	1 (3%)	0 (0%)	1 (1%)

[a] Percentages do not equal 100 because participants could identify as more than one ethnicity.

[b] Percentages do not equal 100 due to rounding.
