# Supplementary material for: iACT4IBD: a randomised controlled trial of a brief online intervention based on acceptance and commitment therapy to improve wellbeing for adults with inflammatory bowel disease
Source: Front Digit Health. 2025 Jun 26;7:1587765. doi: 10.3389/fdgth.2025.1587765 (PMC12241075; doi:10.3389/fdgth.2025.1587765)
Supplement: Supplementary file 3 [file Table3.pdf]

Mental and Physical Health Outcomes of the iACT4IBD Intervention Group at 8-Weeks Follow-up

Outcome Variable	Intervention Group (iACT4IBD, *n* = 13) *n* (%)
**Depression score**	
Normal	11 (85%)
Mild	2 (15%)
Moderate	0 (0%)
Severe	0 (0%)
Extremely severe	0 (0%)
**Anxiety score**	
Normal	8 (62%)
Mild	3 (23%)
Moderate	2 (15%)
Severe	0 (0%)
Extremely severe	0 (0%)
**Stress score**	
Normal	7 (54%)
Mild	6 (46%)
Moderate	0 (0%)
Severe	0 (0%)
Extremely severe	0 (0%)
**Wellbeing score**	
Score of 50% and above	8 (62%)
**Quality of life score**	
Slightly worsened	3 (23%)
Moderately worsened	5 (38%)
Severely worsened	5 (38%)
**Crohn's activity score**	
Remission	1 (33%)
Mild disease	2 (67%)
Moderate disease	0 (0%)
Severe disease	0 (0%)
**Ulcerative colitis score**[a]	
Remission	3 (30%)
Mild disease	2 (20%)
Moderate disease	4 (40%)
Severe disease	0 (0%)

[a] Percentage does not add up to 100% due to one participant not completing this questionnaire.
